# Volatile Organic Compounds as Insect Repellents and Plant Elicitors: an Integrated Pest Management (IPM) Strategy for Glasshouse Whitefly (*Trialeurodes vaporariorum*)

**DOI:** 10.1007/s10886-020-01229-8

**Published:** 2020-10-27

**Authors:** Niall J.A. Conboy, Thomas McDaniel, David George, Adam Ormerod, Martin Edwards, Paul Donohoe, Angharad M. R Gatehouse, Colin R. Tosh

**Affiliations:** 1 School of Natural and Environmental Sciences, Newcastle University, Newcastle-upon-Tyne, NE1 7RU UK; 2 Stockbridge Technology Centre, North Yorkshire, Y08 3TZ UK

**Keywords:** Whitefly, IPM, Tomato, Repellent, Plant elicitor, Volatile organic compound, VOC

## Abstract

The glasshouse whitefly (*Trialeurodes vaporariorum* Westwood) is a polyphagous arthropod pest that is of particular detriment to glasshouse grown tomato (*Solanum lycopersicum*) across temperate regions of the world. Control of whiteflies with synthetic pesticides has resulted in the evolution of resistant genotypes and a reduction in natural enemies, thus highlighting the need for environmentally sound control strategies. Volatile organic compounds (VOCs) offer an environmentally benign alternative to synthetic chemical sprays and this study explored the use of VOCs as insect repellents and plant defence elicitors to control whiteflies on tomato in a commercial glasshouse setting. Limonene in the form of a volatile dispenser system was found to successfully repel whitefly from the target crop and increased fruit yield by 32% during a heavy whitefly infestation. Analysis of tomato herbivore induced plant volatiles (HIPVs) led us to select methyl salicylate (MeSA) as the plant elicitor and application of MeSA to un-infested tomato plants was found to successfully reduce whitefly population development and increase yield by 11%, although this difference was marginally statistically significant. Combination of these two methods was also effective but whitefly abundance in combined plots was similar to the standalone limonene treatment across the course of the experiment. All of the VOC based control methods we used had a negative impact on whitefly performance, with more pronounced effects during the first few weeks of infestation. In subsequent laboratory experiments, we found elevated peroxidase (POD) activity and a significant increase in TPX1 and PR1 transcripts in MeSA treated plants. This led us to deduce that MeSA immediately induced plant defences, rather than priming them. We did however see evidence for residual priming, as plants treated with MeSA and infested with whiteflies produced significantly higher levels of POD activity than whitefly infestation alone. Despite the fact that our treatments failed to synergise, our methods can be optimised further, and the effectiveness of the standalone treatments is promising for future studies. In particular, our repellent limonene dispensers were extremely effective at deterring whiteflies and offer a low economic cost and easy to implement whitefly control option. The methods we have used here could be incorporated into current integrated pest management (IPM) systems, a sustainable approach to pest control which will be central to our efforts to manage whitefly populations under glass in the future.

**Electronic supplementary material:**

The online version of this article (10.1007/s10886-020-01229-8) contains supplementary material, which is available to authorized users.

## Introduction

The glasshouse whitefly (*Trialeurodes vaporariorum* Westwood*)* is a widespread and persistent pest species of many horticultural and ornamental crops, including tomato (*Solanum lycopersicum*). In commercial glasshouses whitefly populations are often managed with biocontrol, typically the parasitoid *Encarsia formosia* (Hanan et al. 2017). However, issues such as delayed efficacy and hyper-parasitism can result in failure, meaning there is still a reliance on synthetic chemical sprays (George et al. 2015). Whiteflies have shown resistance to some of our most important insecticides (Gorman et al. 2007; Wardlow et al. 1976) and the EU has placed restrictions on development of new chemical sprays (Hillocks 2012). Therefore, the need for alternative control methods is clear. Additionally, the human health related impacts of pesticides are widely recognised and major legislative advisory bodies are urging increased use of integrated pest management (IPM) (128/EC 2009; USDA-ARS 2018). IPM systems operate by limiting applications of chemical pesticides (Stenberg 2017) and placing more focus on the use of naturally occurring control agents (Prokopy 2009). IPM as a concept has existed for decades (Stern et al. 1959) and, whilst still under used as a commercial pest control mechanism, is widely regarded as the future of sustainable agricultural pest management. To meet the challenge of ultimately replacing chemical pesticides in the future, novel control components must constantly be developed (Stenberg 2017). Volatile organic compounds (VOCs) are natural products of almost all plant taxa (Vivaldo et al. 2017) and offer an extremely versatile option for incorporation into IPM systems. VOCs are considered environmentally benign alternatives to insecticides and have a proven action against some of our most important insect pests, including whiteflies (Schlaeger et al. 2018). The current study seeks to explore the use of VOCs in the form of an IPM system to control whiteflies on glasshouse grown tomatoes.

Whiteflies are known to respond to subtle changes in their olfactory environment and recognition of volatiles is used in their selection of a suitable host (Bleeker et al. 2009; Darshanee et al. 2017; Shi et al. 2016). Whilst some studies have identified individual VOCs that are repellent to whitefly (Bleeker et al. 2009; Li et al. 2014; Sacchetti et al. 2015), work implementing whitefly repellent VOCs into a commercial IPM set up are extremely limited (Schlaeger et al. 2018). Despite this, we recently had success repelling whiteflies from a tomato crop using limonene, a compound which we identified from the whitefly non-host French marigold (*Tagetes patula L*.; Conboy et al. (2019)). Limonene dispensers were effective under laboratory conditions but were limited in their ability to curb a large pre-existing whitefly infestation in a commercial glasshouse setting. These results indicated that using limonene from the beginning of the growing period could be more effective. In the present study, we decided to use limonene dispensers from the beginning of the growth period to assess whether this could “push” whiteflies from the tomato crop and subsequently increase plant performance. We decided to utilise limonene in the form of a slow-release bottle (as has been demonstrated previously (Conboy et al. 2019; Du et al. 2016)) to allow a constant dispersal rate of the compound. This odour based method of pest control is likely to cause fewer deleterious effects on non-target organisms, and was actually found to be more effective than direct repellent spray application (Du et al. 2016).

VOCs can also be used to induce or optimise plant immune systems; a term previously referred to as “green vaccination” (Luna-Diez 2016). Pest control via the manipulation of plant immune systems is relatively cheap, uses low levels of generally benign plant compounds and can increase a plant’s attractiveness to biocontrol agents (Bruce et al. 2017). Despite the lack of applied research in a commercial agricultural setting (Martinez-Medina et al. 2016), green vaccination still offers tremendous potential for incorporation into IPM systems (Bruce et al. 2017; Luna-Diez 2016). In response to defence eliciting compounds, plants can either directly induce or prime their innate defensive measures (Heil and Ton 2008; van Hulten et al. 2006). Plant priming is a phenomenon whereby the plant enters an induced state of readiness in which the plant will respond more rapidly and effectively to insect attack (Frost et al. 2008; Martinez-Medina et al. 2016). This is a much more attractive method of defence induction to growers as there is little or no energy cost incurred during this primed state, meaning yield will not be impacted if the pest never successfully infests the crop. VOCs have been found to prime plant defences in the form of mixtures (Farag and Pare 2002; Hu et al. 2018) or individual compounds (Erb et al. 2015; Song and Ryu 2018). Other research groups have successfully selected defence-inducing VOCs based on release from infested conspecifics (Erb et al. 2015) and involvement in the plants’ defence signalling pathways (Shulaev et al. 1997; Tang et al. 2015). For tomato, methyl salicylate (MeSA) is a compound that fulfils both these criteria. MeSA is released by tomato in response to herbivory, mainly from sap sucking insects (Ament et al. 2004; Lopez et al. 2012) but also from some chewing insects (James 2003). It is a potent inducer of plant defence (Heil and Ton 2008). It has also been proposed that MeSA could serve as a long distance signalling molecule for the plant (Heil and Ton 2008), as has been previously shown in tobacco (Park et al. 2007). We therefore hypothesised that exogenous application of MeSA to un-infested tomato plants, at concentrations similar to that released by whitefly-infested tomato, would induce a defence response that when the defences abated would confer resistance to a later attack by the glasshouse whitefly. We therefore undertook headspace analysis of whitefly infested tomato plants to confirm the presence of MeSA and quantify emission rates for exogenous application to naïve tomato plants. However, an alternative hypothesis is that MeSA would make tomatoes more vulnerable to whitefly attack by inducing SA induction and reducing expression of JA-induced defences, as shown for *Bemisia tabaci *(Su et al. 2015; Zhang et al. 2013). There is also evidence of possible repellence of other Hemipterans by MeSA in the field (Braasch et al. 2012; Rowen et al. 2017).

Given that the individual components of IPM rarely achieve complete control alone (Hillocks 2012), and since the two different strategies investigated here place performance pressure on pests in different ways, we sought to synergize these two methods for maximum efficacy against glasshouse whiteflies. We sought to produce a level of control greater than the sum of the constituent parts, an important aspect of IPM (Stenberg 2017) by repelling whitefly from a tomato crop with a volatile-based system and then reducing the impact of the lower number of whitefly which subsequently fed on plants by inducing plant defences. Individual IPM components rarely achieve perfect control alone; our proposed mode of action was that the defence elicitor would deal with those insects that make it through the repellence component of the control strategy.

We hypothesised that 1) limonene introduced early in tomato growth would be effective at repelling herbivores; 2) biologically relevant MeSA concentrations would prime tomato defences to reduce subsequent whitefly performance; and 3) the combination of these two treatments would be more effective than the treatments separately. We assessed the efficacy of combined and standalone treatments at reducing whitefly abundance on tomato in a commercial glasshouse setting, measured by assessing adult, nymph, and egg numbers on a tomato crop. Differential effects on these different whitefly stages were not anticipated; reduced adult numbers were expected to have a concomitant reduction on the other whitefly stages. MeSA was selected as the plant elicitor and was sprayed onto plants before introduction to the experimental glasshouse. Limonene was used as the whitefly repellent VOC and was deployed in the form of a slow-release dispenser that was placed alongside plants for the duration of the infestation period. Plants were grown from seed through to harvest in order to assess if our VOC based protection methods could improve yield during a heavy whitefly infestation. Following the glasshouse trial, activity of tomato defence related enzymes and expression of defence related gene transcripts were analysed to characterise resistance shown in the MeSA treated plants. In order to deduce effects on tomato defence signalling, we analysed expression of genes that related to salicylic acid (SA) and jasmonic acid (JA) signalling pathways, in order to detect any effect on relevant defence signalling pathways. In summary, this experiment aimed to assess the potential for VOCs to be used as a component of IPM in commercial tomato production systems and to elucidate any performance effects on the plants and insects involved.

## Materials and Methods

### Whitefly

Whiteflies, *T. vaporariorum*, originated from a lab culture at Rothamsted Research that was first collected in 1960 in Kent on French bean and had subsequently been maintained in a large laboratory population. The insects for both glasshouse and laboratory experiments were taken from a mixed age colony maintained on pre flowering aubergine (*Solanum melongena* “Moneymaker”- Marshalls Seeds Cat. 1020–2017) at 20 °C, 16:8 light/dark.

### Tomato Plant Growth Conditions and Treatments

**Fig. 1:**
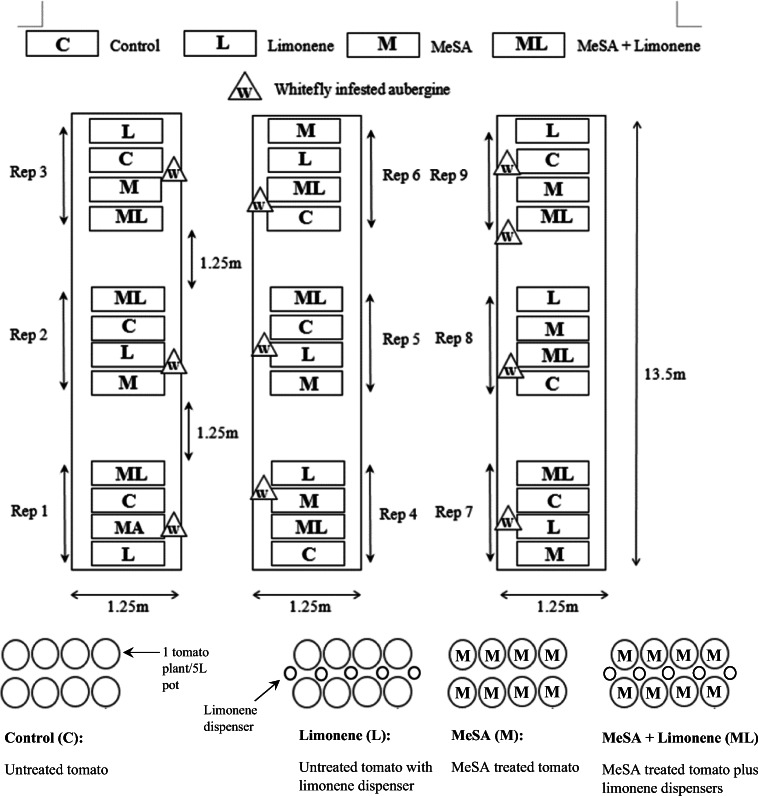
Layout of the glasshouse experiment assessing the impact of repellent volatiles and plant elicitation on whitefly performance. Treatments were arranged in a randomised block design with 9 replicates containing 4 treatments in a random order. The treatments were arranged as shown in the figure above, with heavily infested aubergine plants (labelled in Fig. 1 with a “W”) distributed around the glasshouse as shown

For the glasshouse experiments, ‘Elegance’ tomato plants were grown from seed in standard germination trays in a pest-free propagation glasshouse at Stockbridge Technology Centre (UK) from 1st - 22nd of July 2017. As the first leaves began to appear, half of the 288 seedlings were sprayed with MeSA (Sigma-Aldrich, M6752) dissolved in 50% ethanol at a concentration commensurate with the amount of MeSA we found to be released by whitefly infested tomato plants on each day of infestation (SI. Tab. 1). This concentration was calculated from experiments conducted for this study (SI. Tab1). On day 1 each plant was sprayed with 67.76 μg of MeSA, day two; 60.80 μg, day three; 66.32 μg, day four; 52.84 μg and on day five 143.96 μg. On each day of MeSA application, plants which comprised the Control and Limonene treatments were sprayed with 50% ethanol only. This 5 day spraying regime was based on unpublished work from our group which found that tomato plants exposed to herbivore induced plant volatiles (HIPVs) from whitefly infested conspecifics for 5 days were more resistant to whiteflies (details in SI. Fig. 1; McDaniel (2017)). We aimed to prime plant defences and to do this we considered that for a plant to be primed, the defence response of the plant must return to basal levels before second stimulation from the triggering stress, at which point the plant will respond more effectively to insect attack (Martinez-Medina et al. 2016). We therefore left the MeSA-exposed plants in the same pest free glasshouse for a further 10 days before continuing experimental treatments; whilst definitive estimates of the length of time for defences to abate after a priming event are unknown (Martinez-Medina et al. 2016), this length of time was longer than other studies which have detected functional priming (e.g. Lopez et al. (2012); Ramadan et al. (2011)) and is therefore assumed to be sufficient to have allowed priming to occur in this case.

For enzyme and gene expression assays, tomato seeds (*S. lycopersicum* Mill. var. ‘Elegance’ Cat. E/12/11, Batch 0160685360) were obtained from Monsanto and used for all experiments. Plants were grown from seed in J. Arthur Bowers John Innes no 2 compost in 9-cm-diameter and 8.7-cm-deep pots. ‘Elegance’ seedlings used for the enzyme and genetic assays were grown at a distance of approximately 60 cm from a 400-W Son-T bulb housed in a Harrier HR400SH 400-W lamp under a 16 h light/8 h dark cycle, the temperature regime was 25 °C in the light and 20 °C during the dark period. Treatments applied for the enzyme and gene expression assays were identical, and preparation of treatments was carried out under ambient lighting (16 h light/8 h dark) at 25 °C in the light and 20 °C during the dark period. Treatments for enzyme and gene expression assays consisted of 1) Controls (C): naïve ‘Elegance’ seedlings at stage 14 on the BBCH scale (Klingauf 2001). 2) Methyl salicylate treated (MeSA): as first true leaves emerged, ‘Elegance’ seedlings were sprayed with MeSA at the same frequency and concentration as described above and subsequently left for 10 days. After this treatment course, plants were at stage 14 on the BBCH scale. 3) Whitefly infested (Tv): ‘Elegance’ seedlings at stage 14 on the BBCH scale were placed inside a 30x40cm conicular mesh cage (Watkins and Doncaster, product code: E6090) and infested with 50 adult whiteflies taken from the laboratory culture described under the materials and methods subheading ‘Whitefly’. These plants were infested with whiteflies for 24 h. 4) MeSA treated and whitefly infested (MeTv): This treatment was a combination of MeSA and Tv treatments. After MeSA application, plants were left for 10 days and then infested with 50 whiteflies for 24 h. Following this treatment course plants were at stage 14 on the BBCH scale. 5) Limonene: We also assessed enzyme activity in plants exposed to limonene dispensers to see whether our repellent volatile IPM method had any non-target effects on tomato defences. Eight ‘Elegance’ tomato seedlings at stage 11 on the BBCH scale along with 5 limonene dispensers, arranged in the same way as in the glasshouse experiment (see SI Fig. 2 A for image), were placed inside a 90x60x60cm mesh cage (Watkins and Doncaster, product code: E6098). These plants were left for 10 days until leaf tissue was harvested for enzyme assays, and were at stage 14 on the BBCH scale.

### Glasshouse Experiments

**Fig. 2:**
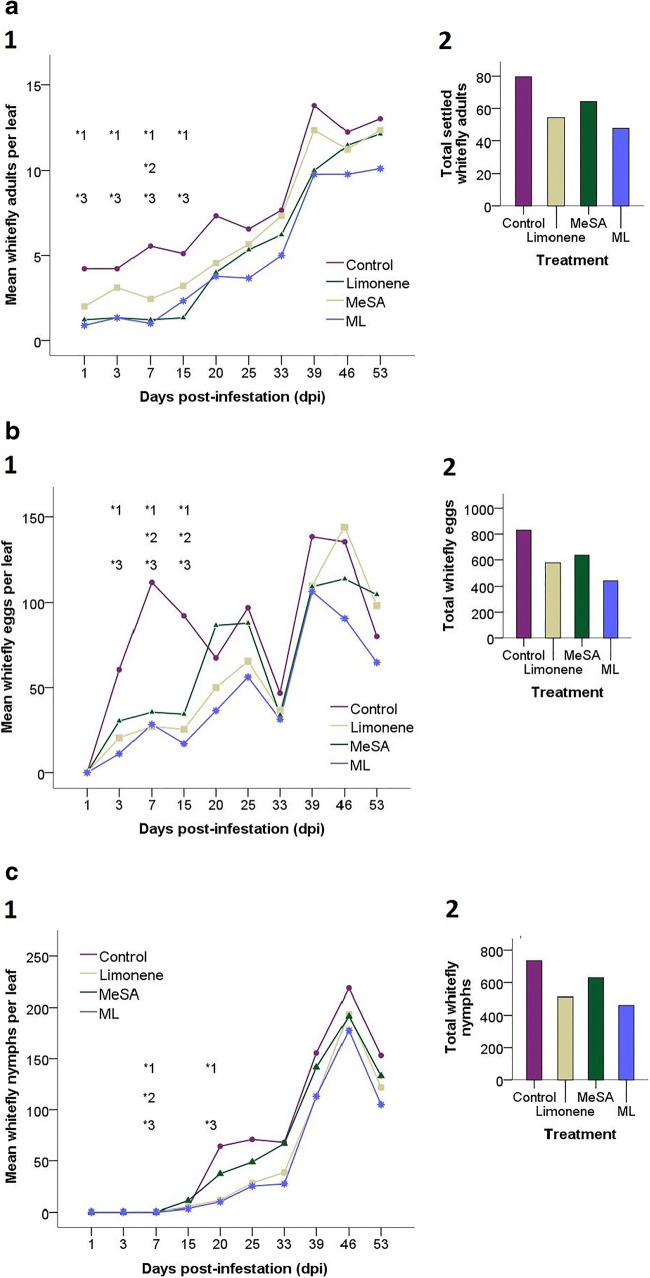
Population development of whiteflies on tomato at all life cycle stages (Settling adults = A, Eggs = B and Nymphs = C) is expressed as average whitefly per leaf (n = 9) across the following treatments: Control = Light grey, Limonene = light blue, MeSA = grey and ML = dark blue. Data were (log+1) transformed and analysed using repeated measures ANOVA’s with Tukey HSD post-hoc tests to compare treatments at individual sampling points (days post infestation (dpi)). Significant observations at individual sampling points are annotated onto the graphs above the sampling point to which this corresponds. The superscript numbers accompanying each significance measure indicate which two treatments are being compared. 1 = Control vs Limonene, 2 = Control vs MeSA and 3 = Control vs ML. (A1) shows the average number of settling whitefly adults per leaf, there was no significant effect of treatment * time but there was a significant effect of treatment following repeated measures ANOVA’s. Post-hoc comparisons revealed there were significantly less whiteflies on Limonene and ML plots at 1, 3, 7, and 15, whereas there were significantly less whiteflies on MeSA plots only at 7dpi. There were also significantly less whiteflies on Limonene plots compared with MeSA plots at 15 dpi. (A2) shows the cumulative total of adult whiteflies counted as settled on sampled leaves over the course of the whole experiment, in order to display trends across the whole growing season. (B1) Shows the average eggs laid per leaf, there was no significant effect of treatment * time, but there was a significant effect between treatments following repeated measures ANOVAs. Limonene and ML treatments had significantly less eggs than control plots at 3dpi, 7dpi and 15dpi. MeSA plots had significantly less eggs than control plots at 7dpi and 15dpi. (B2) shows the cumulative total of whitefly eggs counted on sampled leaves over the course of the whole experiment, in order to display trends across the whole growing season. (C1) Shows the average nymphs (all larval stages) per leaf, there was a significant effect of treatment * time and a significant effect between treatments following repeated measures ANOVA’s. There were significantly less nymphs on all treated plots at 7dpi. At 20 dpi there were significantly less nymphs on Limonene and ML plots compared with controls and significantly less nymphs on ML plots compared with MeSA. (C2) shows the cumulative total of whitefly nymphs counted on sampled leaves over the course of the whole experiment, in order to display trends across the whole growing season. Ninety five percent confidence intervals have been calculated and are available in the supporting information (SI raw data 1), but to aid visualisation they have been removed from the figure

The design of the glasshouse experiments to assess how volatile chemistry and plant defence induction can be combined into an IPM strategy in a commercial setting was as follows. Tomato plants were used at the 3–4 leaf stage (22nd July, day 21 from seed, roughly stage 14 on the BBCH scale) all plants from all treatments were introduced to a 448m^3^ glasshouse (Fig. 1) containing 10 aubergine (*Solanum melongena* “Moneymaker”) plants heavily infested with *T. vaporariorum* (approximately 1000 insects per plant) which were taken from the laboratory culture described in under the materials and methods sub heading ‘Whitefly’. Whilst whitefly were the main focus of our experiment, we allowed natural pest populations to develop without control. *Thrips tabaci* were the only other pest observed in the glasshouse, albeit at insignificant numbers. Throughout the course of the experiment we observed no natural enemies in the glasshouse so any effects on whitefly performance cannot be attributed to parasitism or predation. The plants were arranged into blocks of four different treatments with 8 plants in each treatment and this four treatment block was replicated 9 times (Fig. 1). The control treatment (C) had 8 untreated tomatoes per block, the limonene treatment (L) had 5 limonene dispensers (see Conboy et al. (2019) for details on design) placed along the centre line of 8 untreated tomato (see SI Fig. 2A for image), the MeSA treatment (MeSA) had 8 MeSA treated plants, and the combined treatment (ML) had 8 MeSA treated plants with 5 limonene slow release bottles also. During observations of whitefly development, single fully-expanded leaves were selected by randomising both compound leaf selection and then individual leaflet from a tomato plant (selection of which was also randomised) in each treatment block from each of the 9 replicates. These were then examined in situ for *whitefly* adults and adults of any other insect pests. These leaves were removed and placed in sealed plastic bags, then stored overnight at 4 °C and examined under low power microscopy the next day for whitefly (and other pest) nymphs and eggs. The abundance of whitefly adults settled on sampled leaves at the time of examination, and of eggs (to show levels of oviposition) and nymphs (to show levels of success of hatching) recorded on sampled leaves the next day, were analysed and used as measures of whitefly performance. Sampling was conducted over a 53 day infestation period (22nd July - 13th September) and sampling frequency can be viewed across the x axis of Fig. 2a, b and c.

At the end of the 53 day infestation period, fruit count per plant and fresh weight of total fruits (g) per plant was recorded and average fruit count and weight per plant in each treatment was calculated (*n = 72*) (see SI Fig. 2, B for image of tomatoes at point of harvest). Percentage difference in yield between treatments and controls was calculated with the formula ((Average Treatment Yield – Average Control Yield)/Average Control Yield) × 100. To give indication of fruit quality, one tomato was selected from the lowest fruit cluster of 4 randomly selected plants from each individual treatment block (*n =* 36 total for each treatment) and immediately placed in a plastic bag which was then sealed. These tomatoes were left to ripen in a dark room at ~21 °C for 14 days before being frozen at −20 °C until needed. At which point they were defrosted (12 h), homogenised with a razor blade and assessed for brix soluble solids content using a portable refractometer (PCE-032, PCE-Instruments) (SI. Tab. 2).

### Enzyme Assays

Peroxidases (POD) and polyphenol oxidases (PPO) are defence related enzymes which are often used as chemical markers of induced resistance in members of the *Solanaceae* family (Karban et al. 2003). Both of these enzymes are produced constitutively by tomato but are also induced in response to whitefly feeding (Mayer et al. 2002; McKenzie et al. 2002; Su et al. 2015). We measured the activity of these enzymes to indicate whether induction of plant defences occurred during the glasshouse trial. All five treatments for this experiment (C, L, MeSA, Tv and MeTv) were implemented as described above. PPO and POD activities were measured using modified methods from Thaler et al. (1996) and Rowen et al. (2017). For all treatments, plant tissue apical to the dicotyledonous leaves was removed and flash frozen in liquid nitrogen. This tissue, which ranged from 150 to 300 mg, was homogenised in 1.25 ml of ice-cold K-phosphate buffer (0.1 M, pH 7) containing 2% (*w*/*v*) polyvinylpolyprolidine (Sigma-Aldrich). Subsequently 0.4 ml of 10% Triton X-100 (Sigma-Aldrich) was added to the homogenate, vortexed and centrifuged at 6000 rpm at 4 °C for 15 min. For the analysis of PPO activity, 50 μL of the supernatant was added to 200 μl of 29.2 mM caffeic acid in K-phosphate buffer (0.1 M, pH 8). For the analysis of POD activity, 30 μl of the supernatant was added to 220 μl of 0.3% guaiacol and 0.1% H_2_O_2_ in K-phosphate buffer (0.1 M, pH 8). PPO and POD activities were determined by tracking change in absorbance at 450 nm over 10 min. Activities are presented as change in optical density per minute per gram of fresh weight (Fig. 4).

### Gene Expression

In order to understand the resistance to whitefly shown by MeSA sprayed plants, we analysed expression of three tomato defence related genes LOX1, PR1 and TPX1 (Fig. 5). We assessed activity of PR1 and LOX1 due to their close association with plant defence signalling pathways. LOX-1 encodes a JA regulated enzyme which catalyses the production of 13-hydroperoxy-linolenic acid from linoleic acid (Heitz et al. 1997). The PR-1 gene encodes a pathogenesis related (PR) protein and is commonly used as a marker of systemic acquired resistance (SAR) in across a wide range of higher plants (Chinnasri et al. 2016). We assessed activity of PR-1 due to its close association with the SA defence-signalling pathway (Riviere et al. 2008). After observing high POD activity in MeSA treated plants, we assessed activity of the known POD related gene TPX1 to try further characterise the increased activity of this defensive enzyme. All treatments for this experiment (C, L, MeSA, Tv and MeTv) were prepared as described under the materials and methods subheading ‘Tomato plant growth conditions and treatments’. Total RNA was isolated with Trizol Reagent (Invitrogen, Thermo Fisher Scientific) and purified with PureLink RNA Mini Kit (Invitrogen, Thermo Fisher Scientific), according to the manufacturer’s instructions. Concentrations of the RNA preparations were determined photometrically using a NanoDrop ND-1000 spectrophotometer (Thermo Fisher Scientific). The RNA preparations were stored at −80 *°*C until use. One μg of RNA was reverse transcribed using a reverse transcriptase kit to synthesize cDNA. Primers sequences for actin (reference gene), LOX1, PR1 and TPX1 can all be viewed in SI Table 3, along with the referenced paper which we acquired the sequences from. qPCR was performed with a Rotor-Gene Q (Qiagen), using SensiFAST SYBR No-ROX Kit (Bioline) for 5 min at 95 °C, followed by 35 cycles consisting of 20 s at 95 °C, 30 s at 57 °C and 30 s at 72 °C, then 10 min at 72 °C. All quantifications were normalized to the reference gene actin. The qPCR reactions were performed using three independent RNA preparations from independently grown plants and each RNA sample was run in triplicate for each qPCR run.

### Data Analysis

**Fig. 3:**
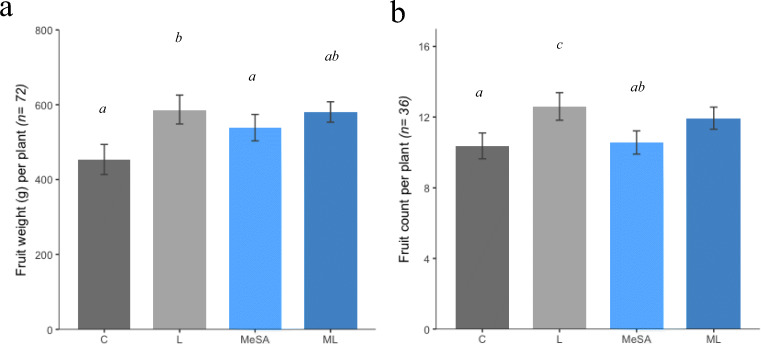
Quantification of plant performance by assessment of fruit weight and fruit count per plant at the end of the 52 day infestation period for each of the four treatments (Control, Limonene, MeSA and ML). Total fruit weight per plant (g) (A) was measured and collated into average fruit weight per plant in each of the four treatments (n = 76). A one way ANOVA followed by Tukey HSD post-hoc tests were used to compare differences between the treatments. Both limonene and ML plots had significantly greater weight of tomatoes per plant than in the control plots. MeSA plots had a greater weight of tomatoes per plant than the control, although this difference was only approached significance. There was also no significant difference seen between the Limonene and ML treatments. On average, the limonene treatment produced a 32% greater weight of tomatoes per plant than the control, the MeSA and ML treatments produced 11% and 21% greater weight of tomatoes per plant than the control respectively. A one-way ANOVA test showed a significant difference in fruit count per plant (B) across the four treatments. Limonene and ML treatments produced significantly more tomatoes than controls but there was no significant difference in tomato numbers between limonene and ML treatments. There was no significant difference in tomato numbers between control and MeSA treatments, but there was significantly more tomatoes in the limonene treatment compared with the MeSA treatment. Error bars on both graphs (A and B) represent 95% confidence intervals. Significant differences between treatments are annotated onto the graph, bars with different letters (a, b or c) denote a significant difference between treatments

**Fig. 4:**
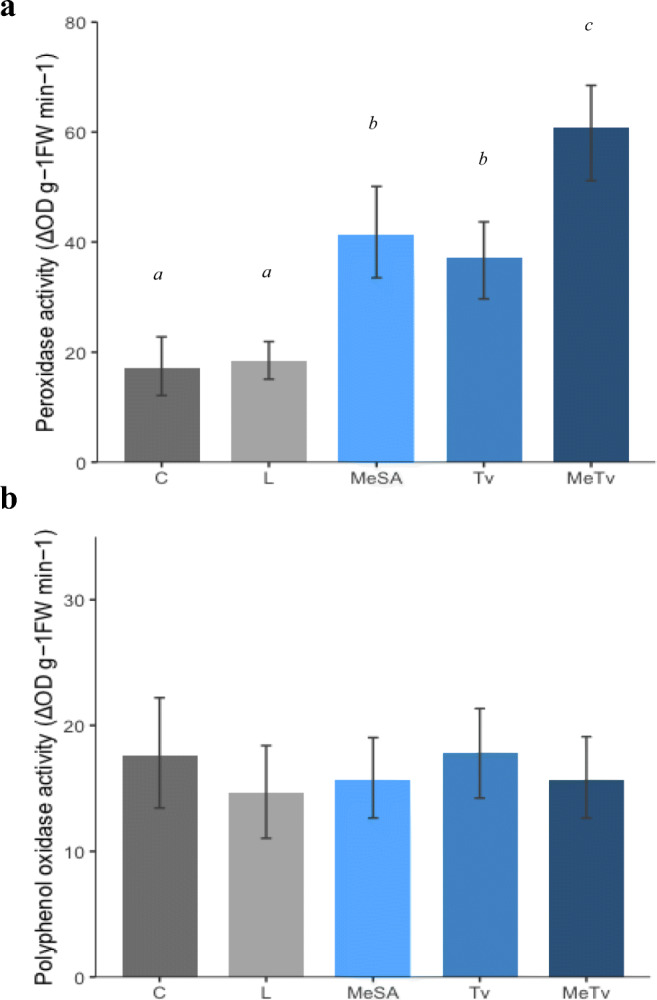
Activity of POD (A) and PPO (B) in control tomato leaf tissue (C) and after exposure to limonene slow release bottles (L), MeSA treatment (MeSA), herbivory from T. vaporariorum (Tv) and MeSA treatment + T. vaporariorum (MeTv) with error bars displaying standard error. Enzyme activity was analysed with one-way ANOVAs followed by Tukey HSD post-hoc tests. There was a significant difference in POD activity (Fig. 4a) between the treatments but no significant effect was observed in PPO activity (Fig. 4b). Tukey HSD post-hoc tests on POD activity revealed no significant difference between Control and Limonene treatments but significant differences between Control and MeSA, Tv, and MeTv treatments respectively. There was also a significant difference in POD activity between MeTV and MeSA and between MeTv and Tv. Significant differences between treatments are annotated onto the graph, bars with different letters (a, b or c) denote a significant difference between treatments

**Fig. 5:**
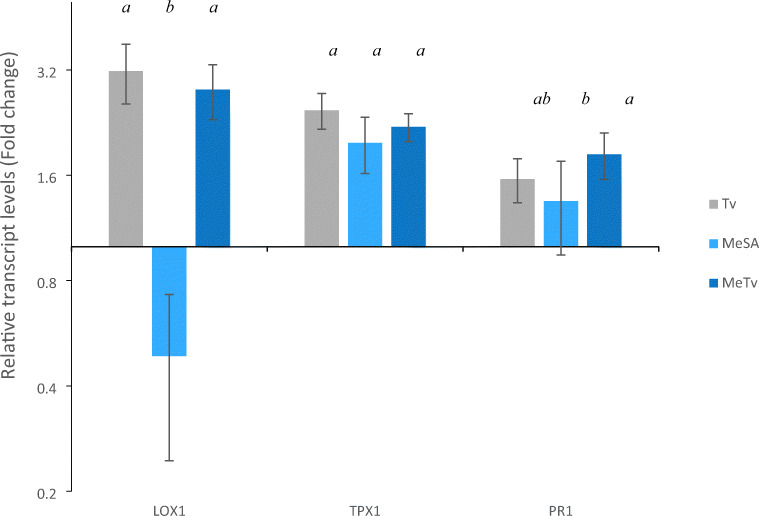
Relative fold change in expression levels of three tomato defence related genes (LOX1, TPX1 and PR1) after MeSA application (MeSA), whitefly infestation (Tv) and MeSA plus whitefly infestation (MeTv). Data for each of the three genes are presented as average (n = 3) relative fold change compared with control transcript levels for each gene. Data analysis was performed on Dcq values and there were significant differences amongst treatments for all three genes following one-way ANOVA’s. The Tukey HSD post-hoc test was used to test differences between the treatments. There were significantly more LOX1 transcripts in the Tv and MeTv treatments, but significantly less transcripts in the MeSA treatment, compared with controls. There was also significantly more LOX1 transcripts in the Tv and MeTv treatments compared with the MeSA treatment. All three treatments had significantly higher amounts of TPX1 and PR1 transcripts compared with controls. There was also a significantly more PR1 transcripts in the MeTv treatment compared with the standalone MeSA treatment. Error bars display 95 % confidence intervals; this was constructed using Dcq values. Significant differences between treatments are annotated onto the graph, bars with different letters (a, b or c) denote a significant difference between treatments

Data for whitefly abundance from the glasshouse trial were (log +1) transformed to meet normality and homogeneity of variance assumptions for statistical analysis. Whitefly abundance at each individual life cycle stage (Fig. 2) was analysed with repeated measures ANOVA’s using the “lmerTest” package in R. Time (sampling date) was used as the repeated measure and treatment was used as the fixed factor. The Tukey HSD post-hoc correction was used to analyse differences between treatments at individual sampling points. Fruit weights/counts (Fig. 3), OD values from the enzyme assays (Fig. 4) and Dcq values from genetic assays (Fig. 5) were analysed using one-way ANOVAs with the base statistics package in R. Tukey HSD post-hoc tests were then used to compare differences between individual treatments. In order to meet normality and homogeneity of variance assumptions, data for POD activity (Fig. 4a) were log transformed.

## Results

### Whitefly Performance in the Glasshouse Experiment

All treatments had a negative impact on whitefly performance at some stage of the 53 day infestation period (Fig. 2). The strongest effects were seen in the early stages of the experiment (first 15 days) where settling rates (Fig. 2a) and oviposition (Fig. 2b) were generally higher on the control plots compared to the other treatments, although there was no significant effect of treatment * time (ANOVA *F*_(27,320)_ = 1.36, *p =* 0.111) but there was a significant effect of treatment (ANOVA *F*_(3,320)_ = 21.40, *p <* 0.001) following repeated measures ANOVA’s. The season totals for settling in Fig. 2a(2) suggest the ML treatment contributes the most to this effect with the least settled whitefly compared to Control, followed by the Limonene and MeSA treatments. There were significantly less settling whiteflies in ML and limonene plots at 1dpi (Limonene, *t =* 2.67, *df =* 320, *p =* 0.039, ML, *t =* 3.32, *df =* 320, *p =* 0.005), 3dpi (Limonene, *t =* 3.88, *df =* 320, *p <* 0.001, M + L, *t =* 3.61, *df =* 320, *p =* 0.002), 7dpi (Limonene, *t =* 4.01, *df =* 320, *p <* 0.001, M + L, *t =* 4.97, *df =* 320, *p <* 0.001), and 15dpi (Limonene, *t =* 4.52, *df =* 320, *p <* 0.001, M + L, *t =* 3.25, *df =* 320, *p =* 0.006). Settling on MeSA plots was only significantly reduced at 7dpi (*t =* 2.76, *df =* 320, *p =* 0.030). There were also significantly less whiteflies on Limonene plots compared with MeSA plots at 15 dpi (*t =* −2.06, *df =* 320, *p =* 0.046).

With respect to oviposition, there was no significant effect of treatment * time (ANOVA *F*_(24,288)_ = 1.49, *p =* 0.066) but there was a significant effect between treatments (ANOVA *F*_(3,288)_ = 10.42, *p <* 0.001) following repeated measures ANOVA’s. Analysis of the season totals for oviposition in Fig. 2b(2) suggest the ML treatment contributes the most to this effect with the least whitefly eggs compared to Control, followed by the Limonene and MeSA treatments. MeSA plots had significantly less whitefly eggs (B) than control plots at 7dpi (*t =* 3.06, *df =* 288, *p =* 0.012) and 15dpi (*t =* 3.44, *df =* 288, *p =* 0.003). Both limonene and ML plots had significantly less eggs at 3dpi (Limonene, *t =* 3.49, *df =* 288, *p =* 0.003, ML, *t =* 3.51, *df =* 288, *p =* 0.002), 7dpi (Limonene, *t =* 3.89, *df =* 288, *p <* 0.001, M + L, *t =* 3.58, *df =* 288, *p =* 0.002) and 15dpi (Limonene, *t =* 4.05, *df =* 288, *p = <*0.001, M + L, *t =* 4.19, *df =* 288, *p* < 0.001). Whilst average settling and oviposition was generally lower (see Fig. 2b at 20dpi and 46dpi for exceptions to this) on protected treatments throughout the experiment, no significant differences were seen between any treatment and control post 20 dpi.

Number of nymphs of all stages revealed a significant effect of treatment * time (ANOVA *F*_(21,256)_ = 2.02, *p =* 0.005) and a significant effect between treatments (ANOVA *F*_(3,256)_ = 12.91, *p <* 0.001) following repeated measures ANOVA’s. Interestingly, the early reduction in oviposition seen between 1dpi and 15 dpi translated to a reduction in nymphs formed on all treatments 7dpi (Limonene, *t =* 3.45, *df =* 256, *p =* 0.003, MeSA, *t =* 3.29, *df =* 256, *p =* 0.006 and ML, *t =* 2.68, *df =* 256, *p =* 0.038; Fig. 2c), showing longer term effects on the whitefly life cycle after this initial 15 day period. The limonene and ML plots also had significantly less nymphs than controls at 20 dpi (Limonene, *t =* 4.30, *df =* 256, *p <* 0.001; ML, *t =* 5.32, *df =* 256, *p <* 0.001) compared with controls, and significantly less nymphs were observed on ML plots compared with MeSA (*t =* 2.88, *df =* 256, *p =* 0.021). Similar to adult and egg abundance, there were consistently less nymphs on all three treated plots across the course of the experiment (Fig. 2c).

In summary, the MeSA plots did not reduce whitefly performance on tomato as effectively or for as long a period as the plots accompanied by limonene slow-release bottles.

### Yield

A one way ANOVA followed by Tukey HSD post-hoc tests were used to compare differences in fruit weight between the treatments. There was a significant difference in average fruit weight per plant between the treatments (ANOVA, *F*_(3,268)_ = 9.85, *p <* 0.001) and all three protected treatments produced more tomatoes than the control (Fig. 3a). Plants from the limonene and ML treatments produced significantly more fruit by weight than the control (Limonene, *t =* 155.80, *df =* 268, *p <* 0.001; ML, *t =* 116.48, *df =* 268, *p <* 0.001) with average fruit weight (g) per plant increasing by 32% and 21% respectively. There was no significant difference seen between the Limonene and ML treatments (*t =* 39.31*, df = 268*, *p =* 0.577). Average fruit weight per plant increased by 11% from the MeSA plots, although this difference was only marginally significant (*t =* 72.69*, df = 268*, *p =* 0.058) following Tukey HSD post-hoc tests.

There was also a significant difference between the treatments when comparing fruit count per plant across the four treatments (One-way ANOVA, *F*_(3,268)_ = 9.99, *p <* 0.001; Fig. 3b). Tukey HSD tests showed that both limonene and ML treatments produced significantly more tomatoes per plant on average compared with controls (Limonene, *t =* 2.85, *df = 268*, *p <* 0.001; ML, *t =* 1.51, *df = 268*, *p =* 0.027), increasing by 28% and 14% respectively. No significant difference was observed in tomato numbers between limonene and ML treatments (*t =* 1.33, *df = 268*, *p =* 0.095). There was no statistically significant difference in tomatoes per plant from the MeSA treatment (*t =* 0.33*, df = 268*, *p =* 0.926), but a statistically significant difference between the limonene treatment compared with the MeSA treatment was observed (*t =* 2.51*, df = 268*, *p <* 0.001). Assessment of Brix percentage soluble solids in tomato fruits (an indicator of vegetable quality) from the four treatments produced no discernible differences following a one-way ANOVA (*F*_(3,140)_ = 1.15, *p =* 0.329, SI Tab. 2).

### Enzyme Assays

Activity of the defence related enzymes PPO and POD were assessed in tomato leaf tissue following exposure to limonene dispensers (L), MeSA application (MeSA), herbivory from whiteflies (Tv) and a combination of MeSA application and herbivory from whiteflies (MeTv) (Fig. 4). Enzyme activity is expressed as change in optical density per minute per gram of fresh weight and statistical analysis compared activity in each of the treatments to control (C) levels. Enzyme activity was analysed with one-way ANOVAs followed by Tukey HSD post-hoc tests. There was a significant difference in POD activity (Fig. 4a) between the treatments (*F*_(4,35)_ = 23.19, *p <* 0.001). Significant differences were observed between Control and MeSA (*t* = 0.41, df = 35, *p* < 0.001), Control and Tv (*t* = 0.36, df = 35, *p* < 0.001) and Control and MeTv (*t* = 0.63, df = 35, *p* < 0.001). There was also a significant difference in POD activity between MeSA plus herbivory (MeTv) and MeSA (*t* = 0.22, df = 35, *p* = 0.044), and between MeTv and Tv (*t =* 0.27, *df =* 35, *p =* 0.008). The limonene (L) treatment had a similar POD activity to controls and there was no significant difference between the two (*t =* 0.06, *df =* 35, *p =* 0.91). Levels of PPO activity (Fig. 4b) showed no significant change between the treatments (ANOVA, *F*_(4,35)_ = 0.47, *p* = 0.752).

### Defence Gene Expression

For all three genes, there was a significant difference in gene expression between the four treatments (C, MeSA, Tv and MeTv) following one-way ANOVAs (LOX1 = *F*_(3, 32)_ = 79.26, *p <* 0.001; TPX1 = *F*_(3, 32)_ = 21.37, *p <* 0.001; PR1 = *F*_(3, 32)_ = 10.91, *p <* 0.001). The Tukey HSD post-hoc test was used to test differences between the treatments.

Whitefly infestation (Tv) significantly increased transcript levels of all three genes compared with respective control levels (LOX1, *t =* −1.66, *df =* 32, *p <* 0.001); TPX1, *t =* −1.29, *df =* 32, *p <* 0.001; PR1: *t =* −0.64, *df =* 32, *p =* 0.002). Tv treatment also displayed significantly higher LOX1 levels than the MeSA treatment (*t =* −2.70, *df =* 32, *p <* 0.001), but displayed no differences in the other two gene transcripts compared with MeSA or MeTV.

MeTV treatment (the combination of MeSA plus whitefly infestation) produced significantly higher transcript levels than controls for all genes (LOX1, *t =* −1.49, *df =* 32, *p <* 0.001; TPX1, *t =* −1.13, *df =* 32, *p <* 0.001; PR1, *t =* −0.87, *df =* 32, *p <* 0.001). MeTV displayed significantly more LOX1 transcripts (*t =* −2.52, *df =* 32, *p <* 0.001) and PR1 transcripts (*t =* −0.44, *df =* 32, *p =* 0.042) than the standalone MeSA treatment, but did not result in any significant differences in any transcript levels compared with Tv, or any differences to other treatments for the TPX1 transcript.

The MeSA treatment significantly downregulated the JA related LOX1 (*t =* 1.03, *df =* 32, *p <* 0.001) and significantly up regulated the SA related PR1 (*t =* −0.43, *df =* 32, *p =* 0.048) compared to controls. The POD-encoding TPX1 was also significantly up-regulated compared to controls following MeSA application (*t =* −0.98, *df =* 32, *p <* 0.001), which is consistent with our experiments on POD activity (Fig. 4a).

## Discussion

In a large scale glasshouse trial we assessed the efficacy of repellent limonene dispensers and plant elicitation with MeSA as combined and standalone control methods for glasshouse whiteflies. All treatments had a negative impact on all three whitefly life cycle stages at some point across the 53 day infestation period, with most of the significant effects between treatment plots and controls seen in the early stages of the experiment (Fig. 2).

Limonene dispensers proved effective at reducing whitefly performance on tomatoes early on in the crop lifecycle, proving our first hypothesis. We propose the insignificant differences seen for settling (Fig. 2a) and oviposition (Fig. 2b) after 20 dpi were most likely due to the comparatively large size of the plants, resulting in the limonene dispensers having less effect on whitefly behaviour as the compound diffused to lower concentrations throughout the glasshouse. Although previous studies have shown MeSA to have effects against insects up to four metres away from dispensers (although at high concentrations; Rowen et al. (2017)) and some pheromone-based dispensers have effects at eight metres (Braasch and Kaplan 2012), the nature of volatile repellence is understood to be dose dependant with higher concentrations having more of an effect (Ben Issa et al. 2016). Maintaining high concentrations of limonene throughout the canopy may prove key in repelling whiteflies in a commercial glasshouse environment, where tomato plants can reach up to 12 ft. in height. An alternative explanation could be that whiteflies habituate to the constant presence of limonene over the course of the experiment. This effect has been shown by Wang et al. (2008) in a diamondback moth/Chinese cabbage system, where the insect habituated to previously-repellent *p*-cymene (extracted from non-host plants) and in some cases increased oviposition in response to the chemical. Further studies would be required to show that a similar effect occurred with whiteflies in our study, but habituation would explain the loss of the protective effect seen early on in the experiment.

Similar to the plots with limonene dispensers, all significant effects on whitefly life cycle stages in MeSA plots were observed in the early stages of the experiment. Defence induction has been shown to be long lasting, even trans-generational (Rasmann et al. 2012), so the longevity of induced resistance is probably not the cause of these short-lived effects on whitefly performance. A potential explanation for this is that the whitefly population in the glasshouse reached a threshold which masked any effects of these defence induced plants: the length of the experiment was such that we would expect the whiteflies to have completed at least one, and possibly two generations, with an accompanying increase in the size of the whitefly population. It is unlikely that growers would introduce plants into a glasshouse with such a large pre-existing pest population and the effects of our treatments may have been revealed more clearly with fewer whiteflies in the glasshouse. The sizes of whitefly populations observed here would certainly have reached the thresholds where pesticides would be applied to a commercial crop; whilst this level varies between insecticides depending on their mode of action, estimates appear to be between four adults per leaf from a random sample (UC ANR Publication 3470, 2013) and one adult per leaflet or five nymphs/ 10 leaflets (Schuster and Smith 2015) hence the population size observed may have been a factor here. Less pronounced MeSA-induced activation of tomato defences could also be as a result of inadequacies in the innate defence responses of this commercial tomato variety, which is known to be inferior to its wild relatives (McDaniel et al. 2016). Identifying aspects of defence which are found wanting in these commercial varieties could prove to be key in optimising the defence responses elicited by MeSA.

Limonene and ML treatments produced a longer lasting effect on whitefly performance compared with MeSA plots with significantly less adults at 15dpi (Fig. 2a) and significantly less nymphs at 20dpi (Fig. 2c) compared with control plots. Considering how plant defences and repellent volatiles challenge whiteflies in different ways, we expected the combined ML treatment to be more effective at reducing whitefly performance than either standalone treatment. Thus the combination of the two control methods should produce an enhanced protective effect (a key tenet of IPM; Stenberg (2017)). However whitefly abundance was similar on both ML and limonene treatments across the course of the experiment and there were no significant differences observed between the two treatments, disproving our third hypothesis. Interestingly, limonene plots produced a slightly higher yield (g) and more tomatoes per plant than the ML plots (Fig. 3), but these differences were not statistically significant. Whilst we cannot attribute any direct fitness costs associated with MeSA application, we can assert that addition of MeSA does not improve yield or decrease whitefly performance when combined with repellent volatile dispensers. Despite this, the MeSA standalone treatment still produced a marginally statistically significantly greater yield than control plots (Fig. 3a), suggesting that there is still value in applying MeSA to elicit tomato defences. Whitefly feeding is known to result in a loss of yield (McKee et al. 2007) and these results show that if left unprotected and exposed to relatively high numbers of insects, tomato can be susceptible to yield loss. The increase in yield (g) from treated plots (Fig. 3) is presumably a consequence of the whitefly performance reduction seen in Fig. 2 and illustrates the effectiveness of our control measures used here. It is possible that the untreated plots could have acted as a refuge for the whiteflies as they were “pushed” from the plots containing the repellent volatile dispensers and/or defence induced plants. Even if this was the case, this still demonstrates the ability of our VOC control measures to manipulate whitefly behaviour and illustrates their potential for incorporation to other IPM systems. In a no-choice situation we predict that limonene would still inhibit whitefly performance as previous groups have shown whitefly to become “restless” when exposed to high concentrations of VOCs (Bernays 1999).

Plants from each of our treatments were assayed for activity of the defensive enzymes PPOs and PODs in order to characterise the resistance to whitefly shown in MeSA treated plants and to ascertain whether limonene dispensers had any non-target effects on plant defence. Plants grown alongside limonene dispensers had levels of PPO and POD activity similar to controls (Fig. 4), suggesting that direct repellent effects of this chemical are responsible for the decreased whitefly performance, as we have shown previously (Conboy et al. 2019). Other groups have found tomato PPOs to be induced by insect herbivory (Bhonwong et al. 2009; Rowen et al. 2017) however we found no significant changes in PPO activity between our treatments (Fig. 4b). Unlike PPO, we found dynamic differences in POD activity across our treatments and POD activity was significantly increased following MeSA application (Fig. 4a). Plant PODs are salicylic acid (SA) related pathogenesis proteins and their ability to scavenge reactive oxygen species (ROS) following herbivory is known to contribute to increased plant fitness (Nath et al. 2016). Other groups have correlated plant PODs with resistance to whiteflies (Taggar et al. 2012; Zhang et al. 2008) and the increase in POD activity observed here could contribute to the increased resistance to whitefly shown in these plants. We cannot claim that our application of MeSA has a priming effect on POD activity as one of the key stipulations of a primed plant is that defences are only transiently or partially induced following the priming stimulus (Martinez-Medina et al. 2016). This is an example of direct defence induction and MeSA in the form of a spray application was found to have a similar effect on POD activity in poplar (Tang et al. 2015). Despite this, it seems that MeSA has increased the capacity of the plant to produce more of this defensive protein as POD activity in MeTv plants was significantly higher than whitefly infested plants (Fig. 4a). Whilst plant defence priming is often associated with preparing for future attack, a primed state may also persist as a residual effect following initial exposure to a stress (Frost et al. 2008). This could be an explanation for the results we have observed here, with MeSA inducing defence responses to an elevated level, followed by stimulation of defences by whitefly infestation resulting in an elevated response. We hypothesised that these enhanced levels of POD in the MeTv treatment could be due to accumulation of peroxidase transcripts following MeSA application, leading to an augmented response upon whitefly infestation. We therefore undertook qPCR of a known peroxidase gene, TPX1, and observed a significant increase in TPX1 transcripts following MeSA application (Fig. 5). Whilst we did not observe the same synergistic effect on TPX1 transcript levels in the MeTV treatment, it is pertinent to consider that multiple genes contribute to peroxidase activity. A more thorough analysis of peroxidase genes could potentially reveal the same synergistic expression pattern. More importantly, these results provide insight into how MeSA elicitation influences tomato antioxidant defences which could be responsible for increased resistance to whiteflies.

Further gene expression analysis was conducted to decipher how MeSA impacts tomato defence signalling pathways (Fig. 5). TPX1 and PR1 transcript levels significantly increased following MeSA application, but there was no increase in TPX1 and PR1 mRNA levels in MeTv plants. Whilst plant priming has previously been characterised by mRNA accumulation (Martinez-Medina et al. 2016), mRNA levels during the priming phase have been shown to be small in comparison to mRNA levels induced by the triggering stimulus (Balmer et al. 2015). The fact that transcript levels are similar in both MeSA and MeTv treatments would suggest that priming has not occurred and that defences have activated with MeSA. Our glasshouse trial illustrates the protective effect of MeSA application and these genetic assays indicate that this increased resistance to whiteflies could be caused by induction of SA related genes, which have been shown to be induced by phloem feeding insects (Smith and Boyko 2007). We propose that spraying of exogenous MeSA has induced this signalling pathway, similar to how methyl jasmonate (MeJA) has been shown to influence the JA signalling pathway (Wu et al. 2008). There is currently a disparity amongst published literature with some groups showing MeSA to induce JA related defences (Rowen et al. 2017) and other groups showing induction of SA related defences (Park 2008). Whilst we can associate MeSA with induction of SA related defences, which may correlate with increased resistance to whiteflies, the way in which MeSA influences plant signalling pathways remains unclear. MeSA caused down regulation of the JA related LOX1, however both Tv and MeTv treatments had significantly higher levels of LOX1 transcripts than controls. JA and SA related defences are often thought to be antagonistic, whereas here we see both SA and JA related genes induced by whitefly feeding. Cross-talk between these two pathways may be responsible for the results observed; whilst this cannot be ascertained from the three genes we analysed, cross talk between the SA and JA pathways is a well-studied interaction, and was observed by Su et al. (2015) in their studies on *Bemisia tabaci*/ tomato interactions. They found that a facultative symbiont secreted in whitefly saliva during feeding, *Hamiltonella defensa*, impaired the tomato defensive response by manipulating cross-talk between the JA and SA pathways. A similar effect may have occurred here: a facultative symbiont may be influencing the tomato defence response in favour of its host. Further studies would be required to confirm this. Finding strategies to moderate or eliminate this interference in the tomato defence response may be an important step in developing IPM components which successfully elicit plant defences, as this relies on the stimulation of the pertinent metabolic processes.

We set out to prime tomato with our application of MeSA, however our subsequent enzyme and genetic assays have revealed that defences were immediately induced with MeSA, disproving our second hypothesis. It has been previously reported that lower concentrations of defence eliciting compounds can induce priming, whereas larger concentrations immediately activate defences (van Hulten et al. 2006). By decreasing the amount of the MeSA it could still be possible to induce priming in tomato, although we predict that this precise nature of delivery could make plant elicitors difficult to use, especially for unexperienced home growers. Despite this, our work does demonstrate that if growers were to introduce plants to a glasshouse with significant pest pressure, application of MeSA could still increase yield. We only tested efficacy of MeSA for protection against whiteflies but other groups have shown MeSA to confer resistance to pathogens (Shulaev et al. 1997) and even caterpillars (Rowen et al. 2017). This broad spectrum resistance could be very attractive to horticulturalists and MeSA could provide an environmentally benign alternative to current synthetic defence elicitors such as benzothiadiazoles, which have been shown to have limitations (Kouzai et al. 2018).

Our work highlights the efficacy of repellent volatile chemistry as a protection method against whiteflies on glasshouse grown tomato and direct repellence of whiteflies using slow-release limonene dispensers is the most attractive whitefly control method investigated here. Limonene dispensers were extremely effective at deterring whitefly from the target crop and offer a cheap, safe, and environmentally benign control method for glasshouse grown tomatoes that could be very attractive to both commercial and domestic horticulturalists. We show that limonene dispensers can effectively “push” whiteflies from a target crop that translates to a more productive yield from these protected plots. We have demonstrated the effectiveness of limonene in an enclosed system, but in an open field situation where pests can potentially be repelled away from the site, limonene could be even more effective. Limonene could also be incorporated into a push-pull system with other volatile dispensers containing compounds attractive to whiteflies, similar to what has been demonstrated with companion plants (Pickett et al. 2014). It could also be combined with other IPM components to form an even more effective system of control. Considering that other pests such as mealybugs (Hollingsworth 2005), mites (Ibrahim et al. 2001) and beetles (Raffa et al. 1985) are repelled by limonene, the potential uses of this control measure could far exceed that of whitefly management. Further studies will be needed to deduce its physiological effect on whiteflies, but the fact that limonene does not seem to cause mortality could be seen as another positive factor. Tolerating and repelling pests rather than completely eradicating them is a more sustainable method of pest control and something which we should look to endorse in our efforts to limit resistant pest genotypes and grow our food sustainably (Peterson et al. 2018).

## Electronic supplementary material

ESM 1 (DOCX 9004 kb)
