# Supplementary material for: Volatile Organic Compounds as Insect Repellents and Plant Elicitors: an Integrated Pest Management (IPM) Strategy for Glasshouse Whitefly (Trialeurodes vaporariorum)
Source: J Chem Ecol. 2020 Oct 27;46(11):1090–104. doi: 10.1007/s10886-020-01229-8 (PMC7677274; doi:10.1007/s10886-020-01229-8)
Supplement: Supplementary file 1 — (DOCX 9004 kb) [file 10886_2020_1229_MOESM1_ESM.docx]

**Supporting Information**

SI Tab 1: Amount of MeSA released per plant at each day of infestation with whiteflies. Data for MeSA μg/g^-1^ FW were calculated by multiplying the ng/μl/g^-1^ FW value by 500 (total volume of sample in μl) and then dividing by 1000 to give to express the value as μg. MeSA per plant was calculated by multiplying the μg/g^-1^ FW value by the total fresh weight of the plant and an average was taken across the three replicates to give a final value of MeSA released per plant (± standard deviation) at each day of whitefly infestation (*n=*3).

| **Days per infestation (dpi)** | **1dpi** | | | **2dpi** | | | **3dpi** | | | **4dpi** | | | **5dpi** | | | |
| --- | --- | --- | --- | --- | --- | --- | --- | --- | --- | --- | --- | --- | --- | --- | --- | --- |
| MeSA ng/μl/g^-1^ FW | 10.400 | 17.562 | 23.376 | 24.239 | 8.4149 | 12.727 | 11.729 | 23.660 | 12.045 | 5.631 | 13.189 | 18.432 | 38.378 | 27.645 | 31.661 |  |
| MeSA  μg/g^-1^ FW | 5.200 | 8.781 | 11.688 | 12.119 | 4.207 | 6.363 | 5.864 | 11.830 | 6.022 | 2.815 | 6.594 | 9.216 | 19.189 | 13.822 | 15.830 |  |
| Total plant FW (g) | 1.97 | 1.96 | 2.00 | 2.01 | 2.03 | 2.00 | 2.21 | 1.96 | 2.22 | 2.11 | 2.15 | 2.12 | 2.22 | 2.20 | 2.21 |  |
| MeSA  per plant | 10.244 | 17.211 | 23.376 | 24.360 | 8.541 | 12.727 | 12.961 | 23.423 | 13.370 | 5.940 | 14.178 | 19.538 | 42.600 | 30.409 | 34.985 |  |
| Average MeSA per plant (μg) | **16.94 (±) 6.57** | | | **15.20 (±) 8.19** | | | **16.58 (±) 5.92** | | | **13.21 (±) 6.84** | | | **35.99 (±) 6.15** | | | |

This experiment utilised information from a previous experiment from our lab group which showed that tomato seedlings exhibited increased resistance to whiteflies after exposure to HIPVs from 4 whitefly infested conspecifics for a period of 5 days, on the basis of significantly lower amounts of whitefly settling and oviposition on the exposed plants than a wide range of other infestation periods (layout of this experiment is shown in SI Fig. 1). We therefore tried to replicate this by applying MeSA to tomato seedlings at concentrations similar to that released by whitefly infested tomato plants. The final value from SI Tab. 1 (average MeSA per plant) was multiplied by four (analogous to HIPV exposure from four plants) and this amount of MeSA was sprayed onto each tomato seedling on the corresponding day. On the first day, 67.76μg of MeSA was applied to each tomato seedling, on day two 60.80μg was applied, day three 66.32μg, day four 52.84μg and on day five 143.96μg was applied.


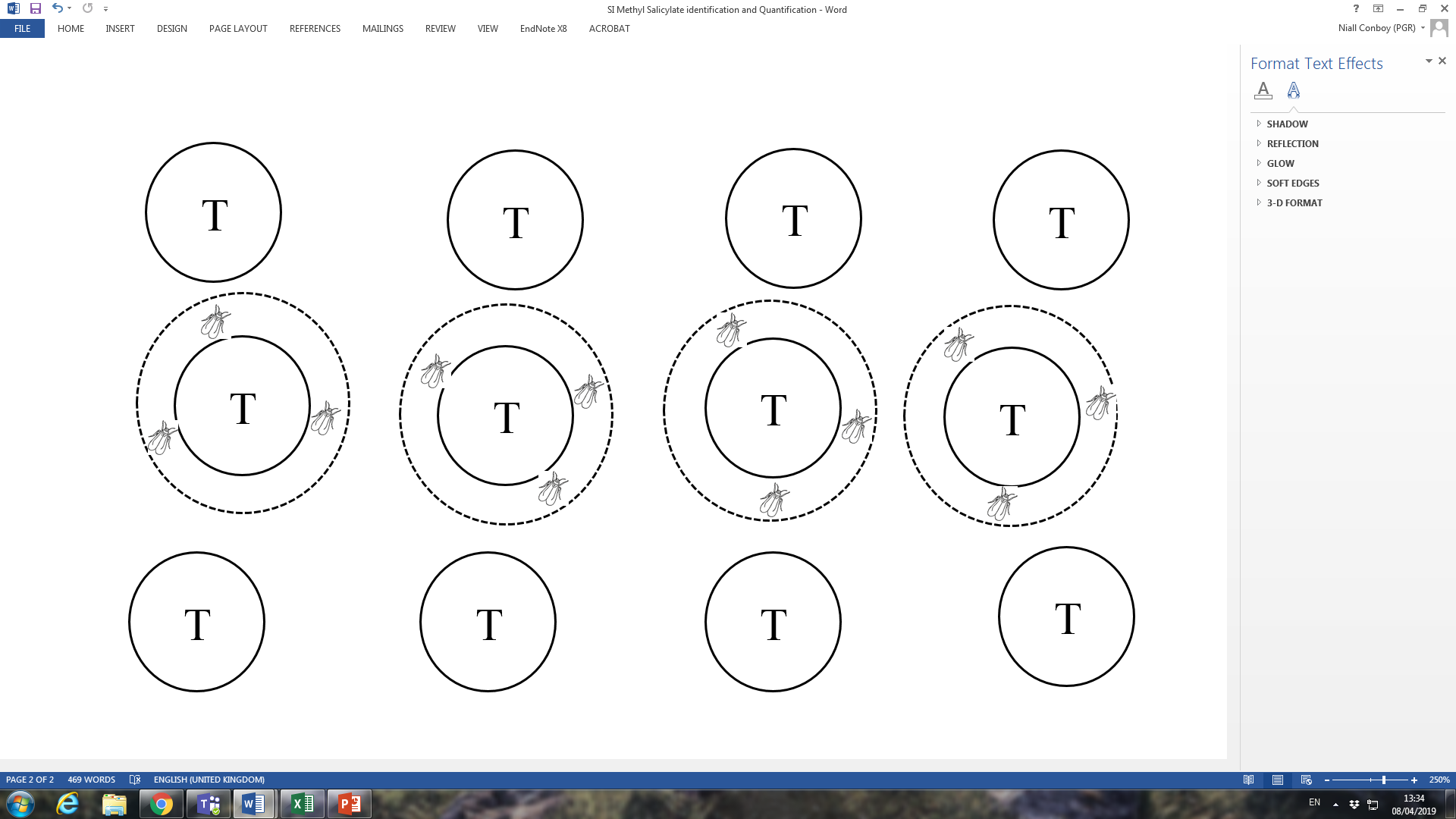
SI Fig. 1: Schematic overview of the experiment conducted by Dr Thomas McDaniel to assess how tomato respond to volatiles from whitefly infested conspecifics (McDaniel 2017). Each ‘T’ represents a tomato seedling, the tomatoes in the centre of the diagram surrounded by a dotted circle indicate plants which were infested with whiteflies. This dotted circle represents a mesh cage which allowed volatiles from these plants to interact with plants outside the cages, whilst also preventing whitefly escape.


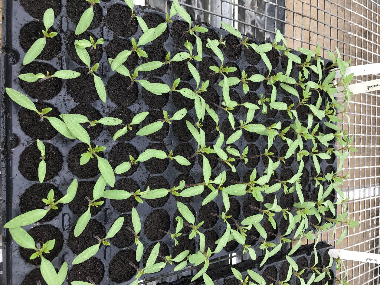

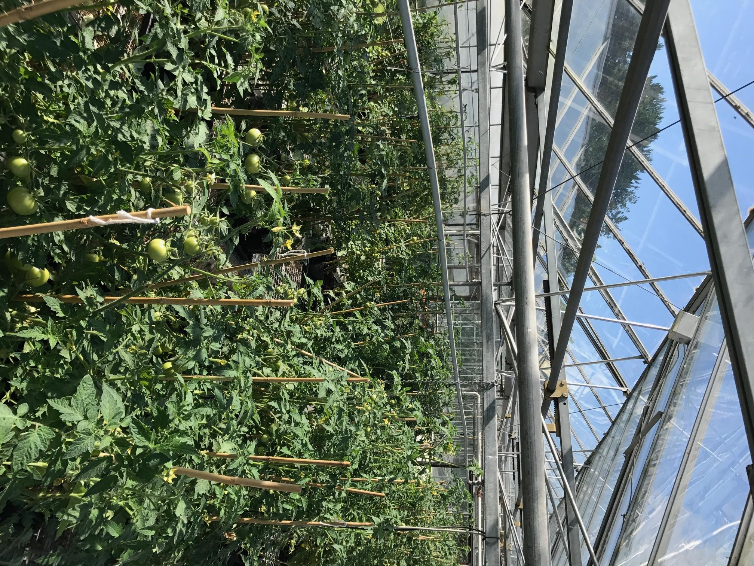

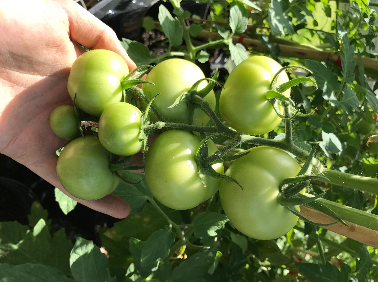

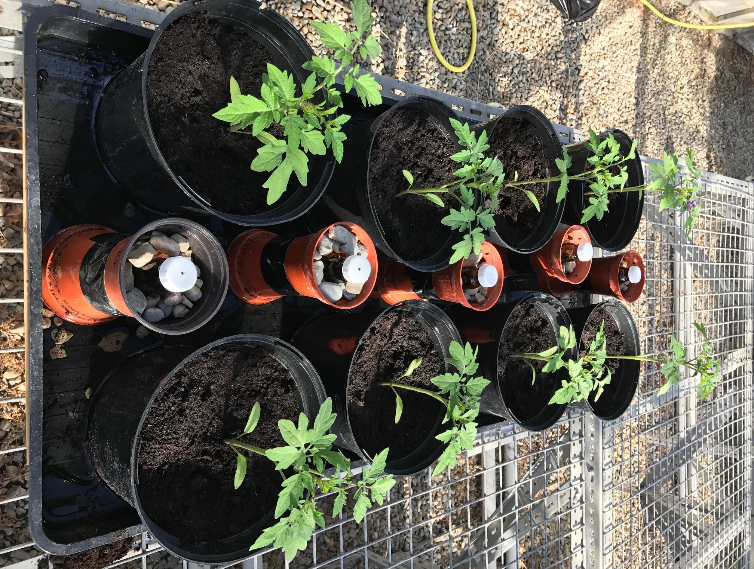


A

C

B

D

SI Fig. 2: A= Overview of how limonene dispensers were distributed amongst tomato plants in the Limonene and ML treatments. B = Typical set of tomato fruits at the point of harvesting. C = Tomato seedlings at day one of MeSA application. D= View across the glasshouse at the final day of sampling.

SI Tab 2: Data for average fruit weight per plant (g), average fruit count per plant and average Brix (%) soluble solids across the four treatments from the glasshouse trial.

| Yield parameter |  |  |  |  |
| --- | --- | --- | --- | --- |
|  | *Control* | *Limonene* | *MeSA*  ***Treatment*** | *ML* |
| Total fruit weight per plant (g) | 483.56 (±) 199.17 *a* | 638.44 (±) 177.97 *b* | 556.25 (±) 167.96 *a* | 600.04 (±) 138.18 *ab* |
| Fruit count per plant | 10.48 (±)  3.41 *a* | 13.43 (±)  3.72 *c* | 10.81 (±)  3.20 *ab* | 12.00 (±)  2.83 *bc* |
| Brix (%) | 3.58 (±)  0.02 | 3.54 (±)  0.02 | 3.60 (±)  0.02 | 3.58 (±)  0.02 |

SI Tab 3: Primer sequences used for gene expression (qPCR) analysis.

| Name | Sequences (5’ → 3’) | Gene | Reference |
| --- | --- | --- | --- |
| TPX1-fwd | TGCAGCATTGACAACACGTA | Peroxidase *(TPX1)* | (El-Gaied et al. 2013) |
| TPX1-rev | TCTTCCCATTTTCTCCATCG |  |  |
| PR1-f | CCTCAAGATTATCTTAACGCTC | Pathogenesis-related (*PR1*) | (Medeiros et al. 2017) |
| PR1-r | TACCATTGCTTCTCATCAACC |  |  |
| LOX-f | GCCTCTCTTCTTGATGGA | Lipoxygenase (*LOX1*) | (Medeiros et al. 2017) |
| LOX-r | GTAGTGAGCCACTTCTCCAA |  |  |
|  |  |  |  |
| Actin-f | CACCACTGCTGAACGGGAA | Actin (*ACT*) | (Medeiros et al. 2017) |
| Actin-r | GGAGCTGCTCCTGGCACTTT |  |  |
|  |  |  |  |
| Mi-1.2-f | \| TCCGAGCTAGATGAGGATGAACA \| \| --- \| | Mi-1.2 | n/a |
| Mi-1.2-r | \| TATCCGGTGAGGTTTCCAGG \| \| --- \| |  |  |

**SI Materials and Methods**

**Air entrainment**

Headspace volatiles of *T. vaporariorum* infested ‘Elegance’ tomato plants were analysed by dynamic air entrainment (Pye Volatile Collection Kit, Kings Walden, Herts, UK) in order to identify potential defence inducing compounds and confirm presence of MeSA. All equipment was washed with Teepol detergent (Herts County Supplies, Herts, UK) and rinsed with acetone and distilled water twice before baking at 180°C for 2 hours. Porapak Q (60/80 mesh, 0.05 g) tubes were eluted with diethyl ether and heated at 140°C for 2 hr under a steam of constant nitrogen to remove contaminants, this process was repeated twice for each tube. Leaflet one of 2-3 week old tomato seedlings was partially enclosed in a glass bell cylinder, due to the slight differences in leaf size fresh weight (g) of the entrained leaf section was recorded after use. The bottom of the cylinder was closed without pressure around the plant stem by using two semicircular aluminum plates with a hole in the centre to accommodate the stem. Charcoal filtered air was pumped in at 1L min^−1^ and drawn out at 800 ml min^−1^ through the porapak Q adsorbent tube in a 5-mm diameter glass tube. The difference in flow rates created a slight positive pressure to ensure that unfiltered air did not enter the system, thus removing the need for an airtight seal around the stem. Plants were entrained for 24 hours from midday onwards. The porapak Q filter tube was eluted with 0.75 ml of diethyl ether (Fisher Scientific, 12347103), providing a 500μl solution that contained the isolated volatile compounds. All samples were sealed in GC vials and stored at -20°C until needed.

**Gas Chromatography (GC)**

GC-FID analyses were carried out using an Agilent 5890 GC. The injection port (280°C) was in the splitless mode and the flame ionization detector was heated to 300°C. The sample (1ul) in diethyl ether was injected by an HP7673 auto sampler and the split opened after 1 minute. Separation was performed on a fused silica capillary column (30m x 0.25mm i.d) coated with 0.25um dimethyl poly-siloxane (HP-5 phase). The GC was temperature programmed from 50°C-310°C at 5°C min and held at final temperature for 20 minutes with Hydrogen as the carrier gas (flow 1ml/min, pressure of 50kPa, split at 30 mls/min).

**Gas Chromatography-mass spectrometry (GC-MS)**

GC-MS analysis of the biological extracts was performed on a Agilent 7890A GC split/split less injector (280°C) linked to a Agilent 5975C MSD (electron voltage 70eV, source temperature 230°C, quad temperature 150°C multiplier voltage 1200V, interface temperature 310°C). The acquisition was controlled by a HP Compaq computer using Chemstation software, initially in full scan mode (50-600 amu/sec) or in selected ion mode (30ions 0.7cps 35ms dwell) for greater sensitivity. The sample (1ul) in diethyl ether was injected by an Agilent7683B auto sampler and the split opened after 1 minute. Separation was performed on an Agilent fused silica capillary column (30m x 0.25mm i.d) coated with 0.25um dimethyl polysiloxane (HP-5) phase. The GC was temperature programmed from 50-310°C at 5°C min and held at final temperature for 10 minutes with Helium as the carrier gas (flow rate of 1ml/min, initial pressure of 50kPa, split at 30 mls/min). Peaks were identified and labelled after comparison of their mass spectra with those of the NIST library if > 90% fit or from their elution order from biochemical literature. Presence of compounds was confirmed by comparison with authentic standards and subsequently semi-quantified using the single point external standard method.

References

El-Gaied LF, Abu El-Heba GA, El-Sherif NA (2013) Effect of growth hormones on some antioxidant parameters and gene expression in tomato GM. Crops & Food 4:67–73. <https://doi.org/10.4161/gmcr.24324>

Medeiros HAd, Araújo Filho JVd, Freitas LGd, Castillo P, Rubio MB, Hermosa R, Monte E (2017) Tomato progeny inherit resistance to the nematode Meloidogyne javanica linked to plant growth induced by the biocontrol fungus Trichoderma atroviride. Sci Rep-Uk 7:40216. <https://doi.org/10.1038/srep40216>
